# Cell culture propagation of foot-and-mouth disease virus: adaptive amino acid substitutions in structural proteins and their functional implications

**DOI:** 10.1007/s11262-019-01714-7

**Published:** 2019-11-27

**Authors:** Veronika Dill, Michael Eschbaumer

**Affiliations:** Institute of Diagnostic Virology, Friedrich-Loeffler-Institut, Greifswald-Insel Riems, Germany

**Keywords:** Foot-and-mouth disease virus, Cell culture, Structural proteins, Virus capsid, Adaptation

## Abstract

Foot-and-mouth disease is endemic in livestock in large parts of Africa and Asia, where it is an important driver of food insecurity and a major obstacle to agricultural development and the international trade in animal products. Virtually all commercially available vaccines are inactivated whole-virus vaccines produced in cell culture, but the adaptation of a field isolate of the virus to growth in culture is laborious and time-consuming. This is of particular concern for the development of vaccines to newly emerging virus lineages, where long lead times from virus isolate to vaccine can delay the implementation of effective control programs. High antigen yields in production cells are also necessary to make vaccines affordable for less developed countries in endemic areas. Therefore, a rational approach to cell culture adaptation that combines prior knowledge of common adaptive mutations and reverse genetics techniques is urgently required. This review provides an overview of amino acid exchanges in the viral capsid proteins in the context of adaptation to cell culture.

**Electronic supplementary material:**

The online version of this article (10.1007/s11262-019-01714-7) contains supplementary material, which is available to authorized users.

## Introduction

Foot-and-mouth disease virus (FMDV) is the etiological agent of the eponymous foot-and-mouth disease (FMD). Infected cloven-hoofed animals present the typical signs of vesicular lesions in and around the mouth and on the feet. The disease affects domesticated as well as wild animals [1]. Although mortality caused by the disease is low, FMD is highly contagious and spreads rapidly. Transmission can occur through contact between susceptible animals, by contaminated air, feed, and water and by fomites [2, 3]. The disease is still common on the African continent, the Arabian Peninsula as well as in South and Southeast Asia [4]. Drastic control measures such as culling of all animals in an infected herd and trade embargoes result in great economic losses to livestock production and farming communities [5]. Therefore, FMD is one of the most important obstacles to agricultural development and international trade in animals and animal products [6]. Inactivated vaccines have been used to eradicate FMD from Europe, South America, and other regions and are still an important tool for control programs in endemic areas as well as for the emergency response to outbreaks in formerly free areas [7, 8]. Commonly, these FMD vaccines are produced by propagation of FMDV in BHK-21 cells [7]. It is known for many viruses, however, that cultivation outside the natural host, for instance during the course of adaptation to a permanent cell line, can result in alterations to the amino acid sequence of viral proteins [9–13]. Especially RNA viruses, such as FMDV, consist of viral populations without a defined genomic sequence but of distributions of related but non-identical genomes (quasispecies). The error-prone genome replication and the missing proof-reading function of the viral polymerase results in high mutation rates, generating a broad variety of genetic and phenotypic variants within the viral population [14]. The adaptation is commonly achieved by repeated passaging in cell culture until the virus grows to high titers [15]. In the case of FMDV, this often leads to an attenuated clinical phenotype in the natural host [13] and can change important physical properties of the viral capsid such as resistance to acids [16–18] and temperature [19]. For vaccine production, the acquisition of mutations that confer an extended receptor tropism is a welcome event that facilitates adaptation of the virus to cell culture. FMDV utilizes cell surface molecules called integrins as a receptor in the natural host and as a primary receptor in cell culture [20–25]. Integrins are heterodimers that consist of an alpha (α) and a beta (β) subunit. In their function as integral membrane proteins, integrins enable cell signaling through conformational changes that facilitate outside-in and inside-out signaling, bind to extracellular matrix (ECM) proteins by recognizing RGD motifs and mediate cell adhesion [26–28]. Four different integrin heterodimers (αvβ1, αvβ3, αvβ6, and αvβ8) can be utilized as cellular receptors by FMDV [21, 22, 24, 29], whereby the virus binds to the cell via the RGD motif and induces internalization [27]. Cell lines commonly used for FMDV propagation present at least one of these integrins to varying extents [30]. Nevertheless, passaging induces the utilization of heparan sulfate (HS) proteoglycans (HSPG) as secondary receptors for the virus [13, 31]. In addition, the Jumonji C-domain containing protein 6 (JMJD6) has been proposed as an additional receptor that enables FMDV to infect cells in an integrin- and HS-independent manner [32]. In any case, adaptation of FMDV to cell culture for vaccine production is laborious, time-intensive, and sometimes not possible at all [33].

There is a wealth of publications that list mutations and adaptations of the viral capsid during the propagation of FMDV in cell culture. Recognizing the mutations that enhance adaptability to cell culture and introducing them by reverse genetics can speed up the process of cell culture adaption and can enable viral growth on cells that are otherwise non-susceptible, with obvious benefits to vaccine development and production. Therefore, this review specifically collates viral capsid mutations related to cell culture without claiming completeness, in the hope of providing a useful overview for virologists, molecular biologists, and biochemists in the field of FMD vaccine development.

## Molecular structure of FMDV

### Classification

FMDV is a member of the family *Picornaviridae* in the order *Picornavirales* [34]. It is the type of species of the genus *Aphthovirus*, whose other members are *Bovine rhinitis A virus* (BRAV), *Bovine rhinitis B virus* (BRBV), and *Equine rhinitis A virus* (ERAV). FMDV is divided into seven antigenically distinct serotypes: O, A, C, Asia-1, and Southern African Territories (SAT) 1, 2, and 3. There have been no documented outbreaks of serotype C since 2004 and it may be extinct in the wild [35].

Nucleotide differences in the genomic region coding for the virus protein 1 (VP1) allow the further division of every serotype into distinct genetic lineages, strains, and geographically clustered topotypes [36, 37]. Serotype A is considered the antigenically most diverse Eurasian serotype, while serotype Asia-1 is thought to be less variable [37].

### Genome organization

**Fig. 1:**
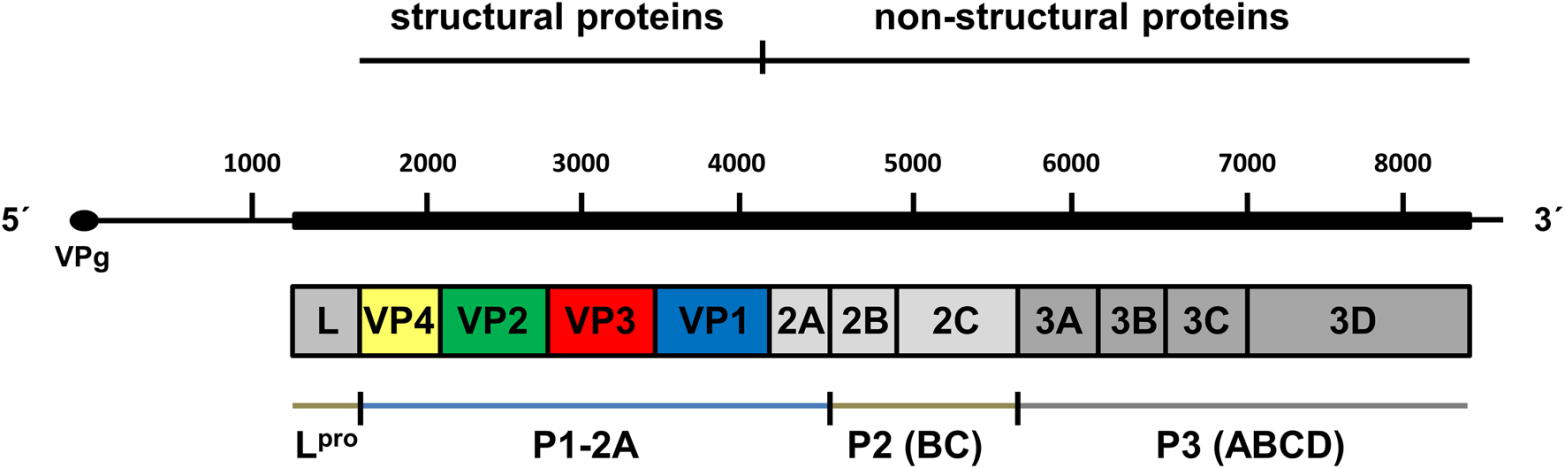
Genome organization of FMDV. The positive-sense single-stranded RNA genome is approximately 8400 bases long and contains a single ORF encoding structural and non-structural proteins. The leader protease (Lpro) and three precursor proteins (P1-2A, P2, and P3) are further cleaved by viral proteases. The figure is based on Jamal and Belsham [38] with modifications

The genome of FMDV consists of a single-stranded, positive-sense RNA (Fig. 1). The viral genome is approximately 8.4 kilobases in length. The 5′ untranslated region (UTR) is covalently bound to a viral genome-linked protein (VPg) [38]. Important structural elements such as the internal ribosome entry site (IRES) are located within the 5′ UTR. Downstream of the 5′ UTR is one large open reading frame (ORF) that encodes a single polyprotein [39]. The polyprotein is co- and post-translationally cleaved into four structural proteins that form the viral capsid and eleven non-structural proteins (NSP) by viral and possibly cellular proteases [6, 14, 38, 39]. Genome replication and protein processing are mediated by the NSP. Another UTR forms the 3′ end of the genome and comprises a stem-loop structure of approximately 100 nucleotides followed by a poly-A tract [39].

### Virion structure

**Fig. 2:**
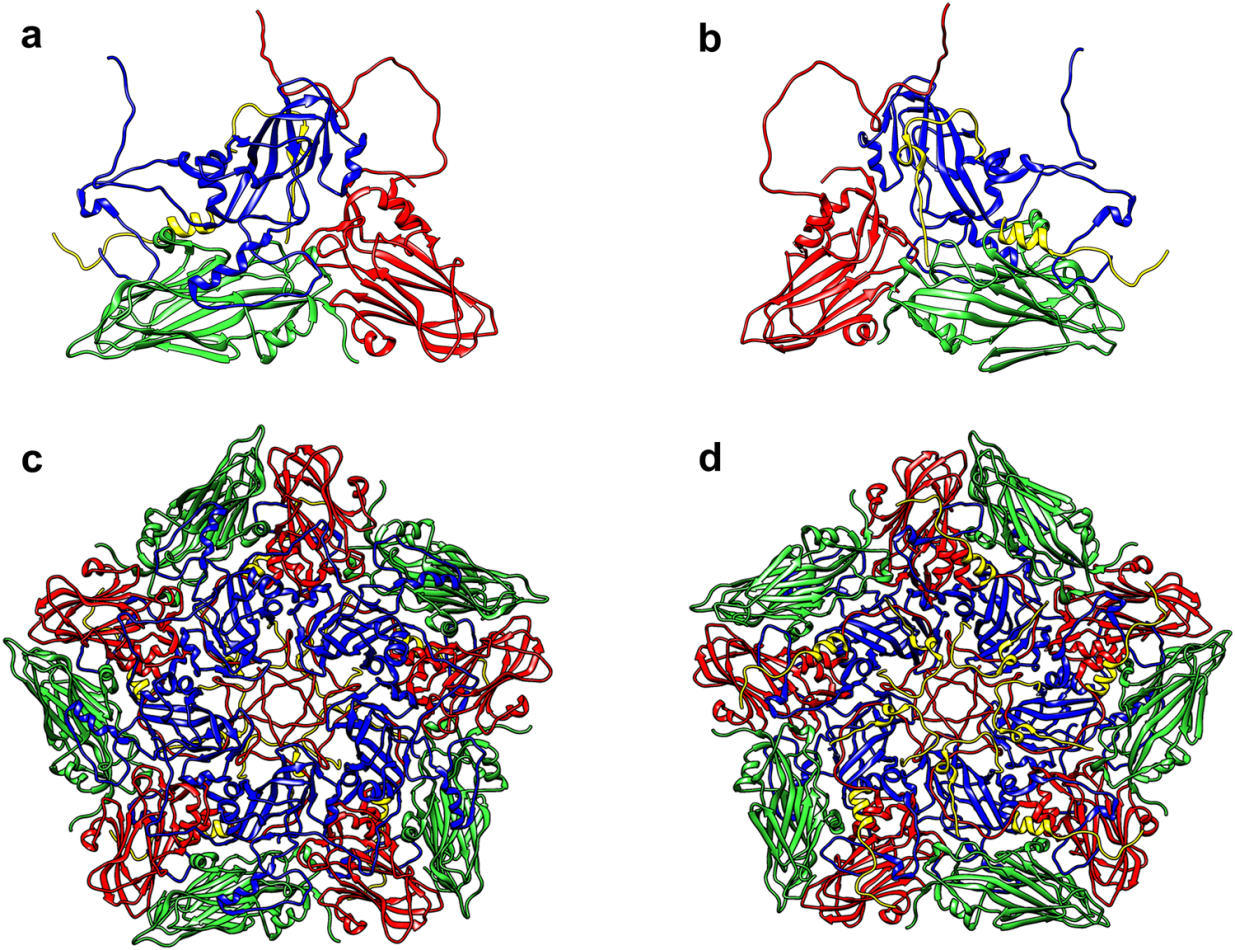
Structural proteins of FMDV and their physical arrangement. A ribbon diagram of the four capsid proteins VP1-4 forming a biological protomer (**a**, **b**) as well as their combination to make a pentamer (**c**, **d**) is shown using the standard color convention: VP1 blue, VP2 green, VP3 red, and VP4 yellow. Because the fourth viral protein VP4 is located on the interior aspect of the viral capsid, the protomer and pentamer are each shown from the outside (**a**, **c**) and from the inside (**b**, **d**). The figure was made using the X-ray crystal structures of FMDV serotype O (1FOD) [49] as template, edited with UCSF Chimera [76] and the embedded MSMS package [77]. Chimera was developed by the Resource for Biocomputing, Visualization, and Informatics at the University of California, San Francisco, USA (supported by NIGMS P41-GM103311)

The viral particle is a spherical icosahedron with a diameter of approximately 25 to 30 nm and no lipid envelope. The surface of the virion is smooth, unusual among picornaviruses [40, 41]. Four proteins, namely virus protein (VP) 1 (1D), VP2 (1B), VP3 (1C), and VP4 (1A), form the viral capsid. While VP1, VP2, and VP3 are exposed at the outer surface and exhibit a high level of variability, the fourth VP is internally located and is the most conserved protein of the viral capsid [40, 42] (Fig. 2).

**Fig. 3:**
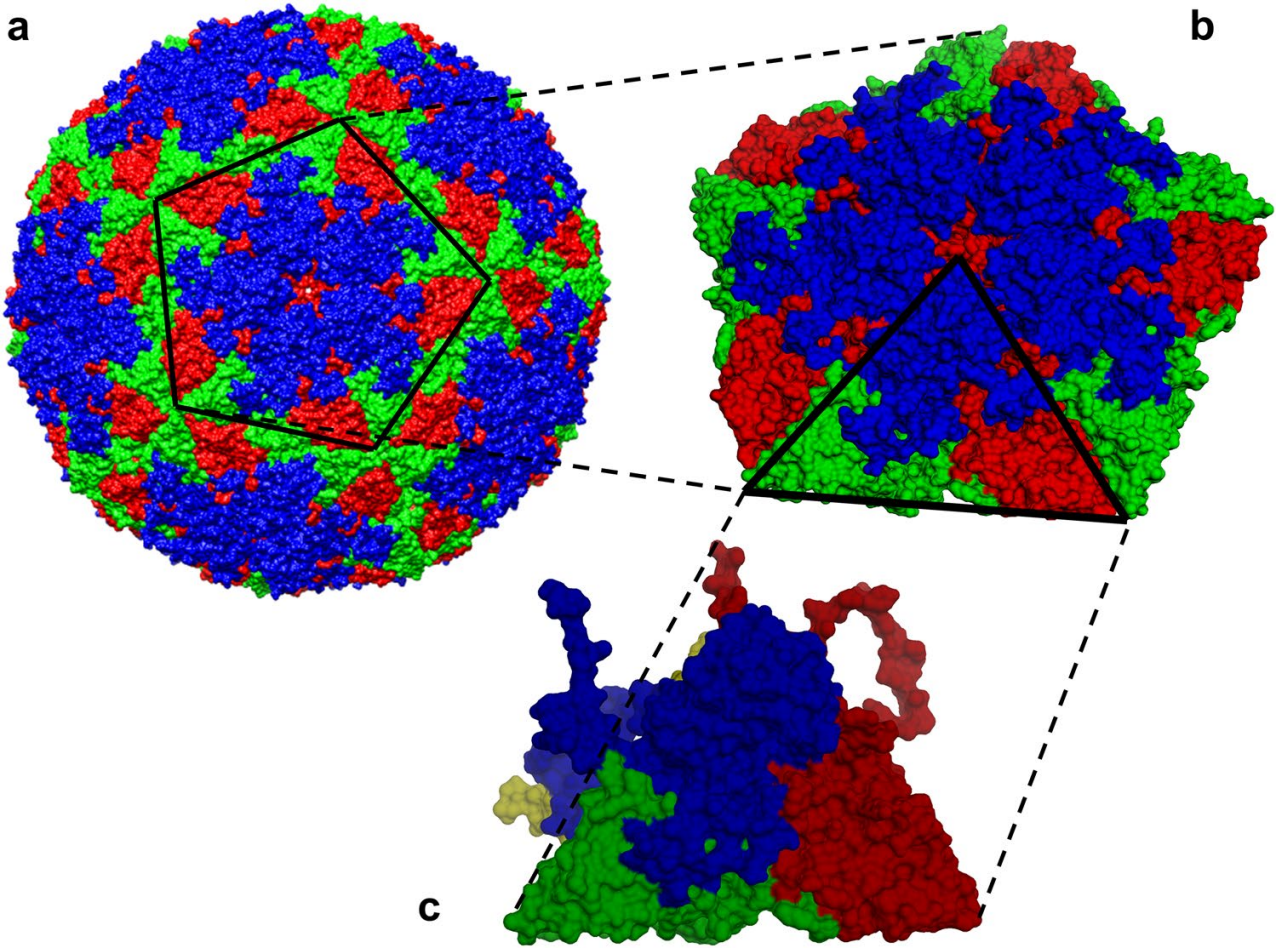
Icosahedral structure of the FMDV particle. The assembled virus particle (**a**) is comprised of 12 identical pentameric subunits (**b**). Each pentamer contains five identical protomers (**c**) that are formed by the outer capsid proteins VP1 (blue), VP2 (green), and VP3 (red) exposed on the outer surface, while VP4 (yellow) is located inside. The figure was created using UCSF Chimera [49, 77] and the X-ray crystal structures of FMDV serotype O (1FOD) [49]

Each of the surface-exposed VPs is formed by a beta-sandwich consisting of eight single strands labeled B, I, D, G, C, H, E, and F and seven connecting loops that are named after the adjacent beta-strands [43]. While the BIDG lamella is drawn together in the inner capsid, the CHEF strands as well as the associated loops are exposed on the outer capsid surface [41]. To make up the viral capsid, heterooligomeric protomers are built from one copy of each structural protein originating from the same P1-2A precursor molecule. Five identical protomers combine into pentamers and the whole capsid is formed by 12 identical pentamers or 60 identical protomers [14, 41] (Fig. 3).

A short motif of three residues (arginine, glycine, and aspartic acid; RGD) at the apex of the GH loop of VP1 is highly conserved, likely due to its important function in binding integrin molecules on the host cell surface [14, 44]. The adjacent residues, however, are highly variable and constitute an important antigenic site [6, 43].

Another unique feature of FMDV particles among the picornaviruses is a pore at the fivefold symmetry axes that permits the entry of small molecules, resulting in the viral capsid with the highest density among the picornaviruses [6, 40]. In total, three kinds of particles can be observed: firstly, the intact FMD virion including the RNA genome, referred to as the 146S particle, which is an essential component of vaccines to provoke a protective immune response [45]; secondly, 75S particles, which are empty capsids without viral RNA. These particles have the same immunogenicity as 146S particles but are less stable in nature; and thirdly, 12S particles, which result from capsid dissociation into pentamers and are only poorly immunogenic [45].

### Capsid proteins

When compiling and comparing previously published observations of amino acid variation, it is essential to keep in mind that three of the four capsid proteins of FMDV can vary in length between different serotypes and strains due to insertions and deletions of amino acids [42]. It is therefore preferable to refer to residues by their position within each viral protein, and not within the polyprotein as a whole. Even then, some ambiguity remains, and care must be taken to positively identify cognate residues between strains and serotypes.

#### Virus protein VP1

**Fig. 4:**
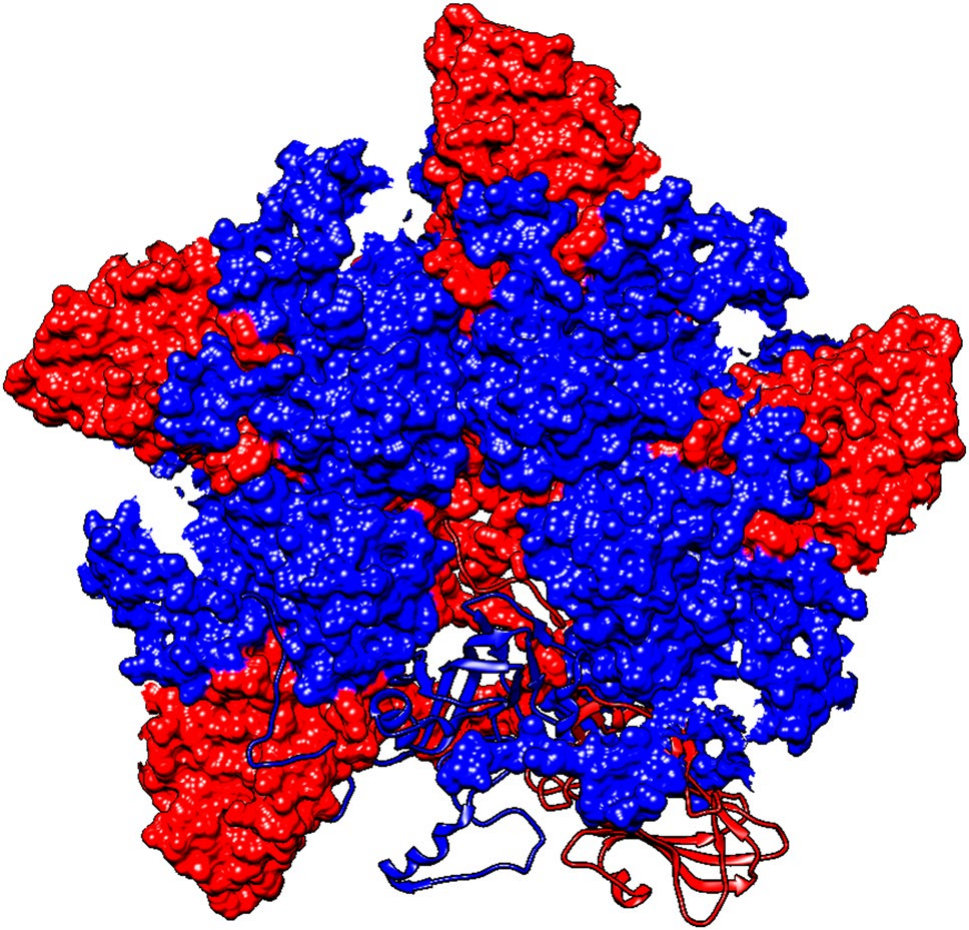
Schematic arrangement of VP1 and VP3 with clockwise overlapping of VP1 and VP3 by the C-terminus of VP1. Chain 1 of VP1 (blue) and VP3 (red) is represented as a ribbon diagram, while chains 2–5 are presented as solid surfaces. The figure was produced using UCSF Chimera and the X-ray crystal structures of FMDV serotype O (1FOD) [49, 76, 77]

VP1 has the highest variability among the capsid proteins, with 74% of its residues being variable [41, 42]. There are three distinct antigenic sites. Site 1 contains the mostly invariant [42] RGD motif at the apex of the GH loop and the highly variable residues around it. The C-terminal residues form site 2 [40], whereas the antigenic site 3 is located in the BC loop (residues 43–45 and 48) [46–48]. The length of VP1 is very variable between serotypes and ranges between 207 and 219 aa, due to insertions or deletions mainly in the region around the GH loop [42, 49]. Unlike other picornaviruses, the C-terminus of FMDV VP1 extends clockwise over VP1 and VP3, filling the depressed surface of the capsid and creating the smooth appearance of the virion [40, 41] (Fig. 4).

**Fig. 5:**
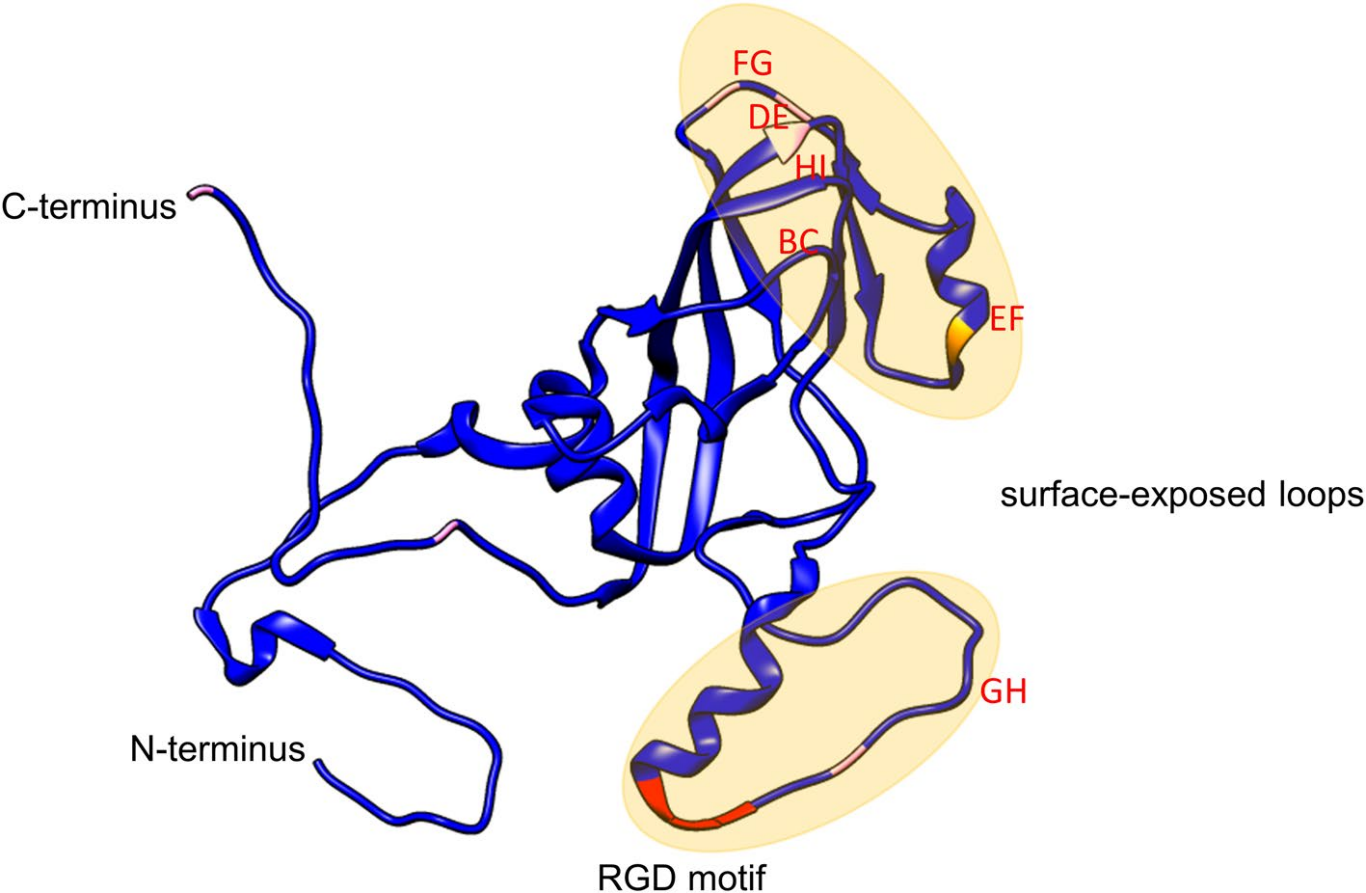
Ribbon diagram of VP1. The protein map is constructed from FMDV serotype O (PDB: 1FOD) [49] with the UCSF Chimera package [76]. The conserved RGD motif is marked in red, while the regions of the protein where amino acid changes occur most often are marked in pink. Residue 95 (close to the EF loop) is marked in orange. The surface-exposed loops of the protein (highlighted by orange ellipses) are labeled in red according to Fry et al. [48]

Because of the high variability of this protein, there is a rich variety of singularly reported amino acid exchanges (see Table 1), but there are certain substitutions that are described more frequently and for different serotypes. These are located at residues 83, 108, 110, 142, 194, and 210 and will be discussed in more detail below (see also Fig. 5).

**Table 1:** Summary of reported amino acid substitutions in FMDV VP1

FMDV type	Virus protein	Amino acid substitutions	References
SAT1, A	VP1	Y18H	[15, 50]
SAT1, A	VP1	T25A, Q25R	[50, 51]
SAT2	VP1	M28V	[50]
SAT1	VP1	A33T	[50]
C/O	VP1	K41E/N, T	[52–54]
O	VP1	K45Q	[54]
C, Asia-1	VP1	D46E, K46G	[53, 55]
SAT1, Asia-1	VP1	R49K, Q49K	[50, 55]
Asia-1, A	VP1	S58A, Q58K	[51, 55]
Asia-1	VP1	H59R	[55]
Asia-1/SAT2	VP1	A64K/G	[50, 55]
A	VP1	L66V	[15]
SAT 1	VP1	A69G	[50, 56]
O	VP1	Y72C	[57]
Asia-1	VP1	E77K	[55]
A	VP1	R81C	[58]
Asia-1, C	VP1	T83A	[33, 59]
**O, SAT2**	**VP1**	**E83K**	[50, 52, 54, 60–63]
SAT 1	VP1	E84G	[56]
SAT2	VP1	Q85R	[50]
SAT1	VP1	K86Q	[50]
*A, O*	*VP1*	*E95K, E95A*	[30, 52, 54, 64]
O, A	VP1	T96A, S96L	[30, 57, 64]
SAT2	VP1	R98T	[50]
**O, Asia-1**	**VP1**	**H108Y, Q108R/R108Q, L108K**	[33, 51, 55, 63]
**Asia-1, A, SAT1**	**VP1**	**R110Q/Q110R, Q110K, N110H/K**	[33, 50, 51, 55, 56, 65]
SAT1	VP1	N111K	[50]
SAT 1	VP1	G112R/D	[50, 56]
A	VP1	T120N	[15]
O	VP1	S134C	[60, 61]
O	VP1	G137D	[57]
C	VP1	A138P/D	[53]
C, O	VP1	S139R/G/N/I, S139R	[53, 54]
Asia-1	VP1	S141P	[55]
**Asia-1, O, C, A**	**VP1**	**R142Q/P, T142A/N142S, G142E**	[51, 53, 55, 57, 63]
A, C	VP1	T143K, D143G	[53, 58, 66]
C, O	VP1	L144V/S, V144A	[53, 54]
A, C	VP1	G145D, A145V	[30, 53]
C	VP1	H146R	[53]
C	VP1	L147P	[67]
Asia-1, C	VP1	V148D, T148K	[55, 59]
C	VP1	T149M	[53]
A	VP1	L150P/R	[30, 64]
O	VP1	A152T	[57]
O	VP1	Q153P	[57]
Asia-1, A	VP1	S154D, V154A/I154N	[15, 30, 68]
SAT1, A	VP1	K157A, Q157R	[50, 51]
O	VP1	T158A	[54]
SAT2	VP1	E161K	[50]
O	VP1	I168V	[57]
SAT2	VP1	Y169H	[50]
SAT2/O	VP1	T171A/P	[50, 63]
O	VP1	T174F	[63]
SAT1	VP1	E177Q	[50]
SAT1	VP1	V179E	[50]
A	VP1	L191S	[15]
Asia-1	VP1	L192P	[55]
**A, SAT2, C**	**VP1**	**E194K, F194L, G194D**	[50–52, 59]
A	VP1	E196K	[51]
C	VP1	H197R	[53, 66]
A	VP1	D198G	[69]
O	VP1	A199T	[57]
C	VP1	P200Q	[66]
A	VP1	H201R	[51]
Asia-1	VP1	E202K	[33]
A	VP1	Q203R	[51]
A, SAT1	VP1	I206V, K206R	[15, 50]
SAT2	VP1	V207A	[50]
**O, A, SAT1**	**VP1**	**K210E, N, R**	[50, 52, 60–62]
O	VP1	L212S	[54, 57]
A	VP1	L213S	[15]
A	VP1	S226F	[15]

Where different amino acid exchanges at one position have been reported for different serotypes, they are separated by commas. If more than one exchange has been described for a serotype, these are separated by forward slashes. Frequently reported amino acid exchanges are given in bold. The JMJD6-receptor-associated capsid protein mutation is given in italics

Residue 95 is located at the interface between two VP1 proteins at the fivefold axis of the virus particle and interacts with the C-terminus of the Jumonji C-domain containing protein 6 (JMJD6) [52, 64]. A substitution of glutamic acid with lysine at this position allows the virus to infect cells in culture in an integrin- and HS-independent manner [30, 52, 64].

Residue 210 is located at the C-terminus of the mature protein. The most commonly described amino acid exchanges replace a positively charged lysine with a glutamate (the anion of glutamic acid) with a single negative charge, but its replacement with an uncharged asparagine or a positively charged arginine has also been observed [50, 52, 60–62]. Amino acid exchanges at position 210 inhibit the cleavage of the VP1-2A product [60–62]. They have been reported for FMDV serotype O, serotype A, and SAT 1 and are always linked to the E83K substitution, also in VP1 [50, 52, 60–62]. This replacement of a negatively charged glutamate with a positively charged lysine is the only exchange ever observed at residue 83. Residue 83 is situated within the DE loop at the outer surface of the capsid. Unlike exchanges at residue 210, those at position 83 can also occur in combination with exchanges at positions 108, 110, 142, and/or 194 [33, 50, 63]. They seem to provide a selective advantage for virus propagation in BHK cells.

Amino acid exchanges at position 108 and 110 also often occur in combination, either with each other or with (additional) exchanges at position 142 or 194. Usually, exchanges at residues 108 and 110 cause an increase of the overall net positive charge at the fivefold axis of the particle [33, 51]. Positioned in the FG loop of VP1, these changes allow integrin- and HS-independent binding of cells as well as enhanced virus propagation in BHK cells [33, 51, 55]. Additionally, the loop made up of residues 84–115 represents a further antigenic site for Asia-1 strains, so that variations in this area could change the overall antigenicity of the virus particle [55].

Residue 142 lies within the GH loop close to the RGD motif and modulates the spatial orientation of the GH loop depending on the amino acid at this position [63]. Substitutions at residue 142 have been reported for all Eurasian FMDV serotypes [51, 53, 55, 57, 63]. These substitutions are highly variable and differ depending on the cell line in which the virus was cultured [51, 55, 63].

Another very variable residue is found at position 194. This residue is close to residues 195–197 that make up one of the walls of the heparan binding site [48]. Amino acid exchanges toward a positively charged amino acid at this position (E194K), predominantly seen in serotype A isolates, are therefore associated with the acquisition of HS as the cellular receptor [51, 52]. The exchanges at that position that have been described for serotypes C and SAT2, on the other hand, do not fit that explanation [50, 59]. Surprisingly, the amino acid exchanges that allow HS binding in cell culture (E196K, H197R) do not seem to be more frequent than other unique mutations reported during culture adaptation [51, 53, 66].

**Fig. 6:**
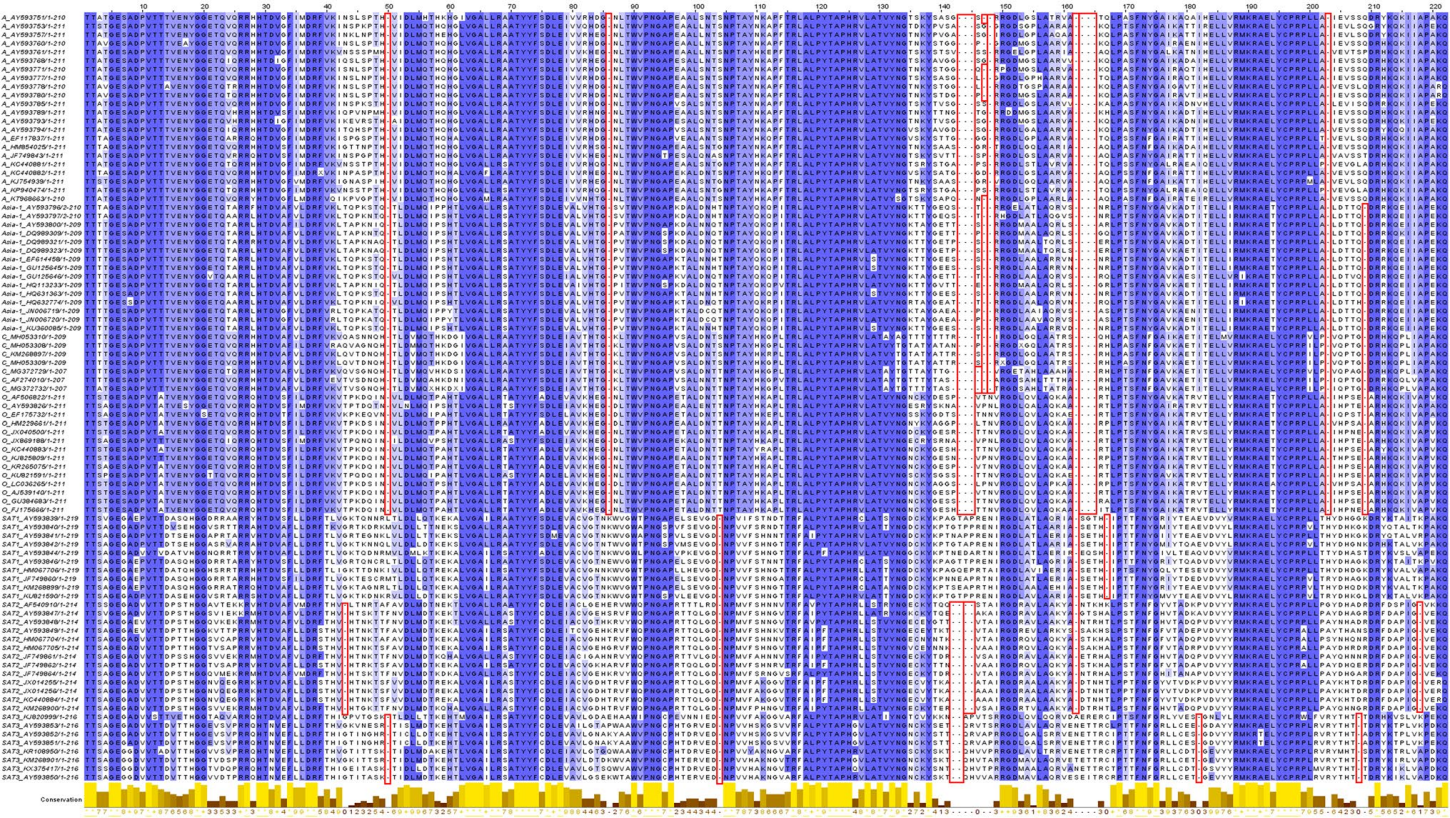
Protein alignment of aa sequences of all seven FMDV serotypes. Nucleotide sequences of the coding region of VP1 were retrieved from the GenBank database (https://www.ncbi.nlm.nih.gov/nucleotide/). Accession numbers are provided directly in the figure. Nucleotide sequences were translated into protein sequences by ExPASy [78]. Protein alignments and visualization were performed with Jalview [79]. Red boxes indicate positions where variations in the sequence lengths appear. The blue coloring indicates the conservation at a position, with darker shades of blue indicating higher degrees of conservation

A protein alignment with several isolates of each FMDV serotype is shown in Fig. 6. The high diversity of VP1 amino acid sequences is evident in the alignment, with only a few regions conserved between serotypes. Isolates of FMDV serotype A had a VP1 of 210 or 211 aa in length, while strains of serotype Asia-1 had 209 or 210 aa and serotype C had 209 or even 207 aa. Serotype O consistently had a VP1 of 211 aa in length, whereas the VP1 of SAT1, SAT2, and SAT3 comprised 219 aa, 214 aa, and 216 aa, respectively. The differences in the length of VP1 are caused by deletions at positions 43, 50, 86, 104, 142–148 (upstream of the RGD motif), 162–165, 167, 182, 203, 208–209, and 218.

Before introducing any of the aa substitutions given in Table 1 by genetic engineering, it is necessary to identify the equivalent residue in the target isolate. The alignment shown in Fig. 6 can be used as a starting point. FASTA files of the alignments are provided as a supplemental to the online version of the article. With a suitable viewer software (such as the NCBI Multiple Sequence Alignment Viewer, https://www.ncbi.nlm.nih.gov/projects/msaviewer/), these files can be used to quickly and easily identify the cognate residue for each of the positions listed in the table across all serotypes.

#### Virus protein VP2

**Fig. 7:**
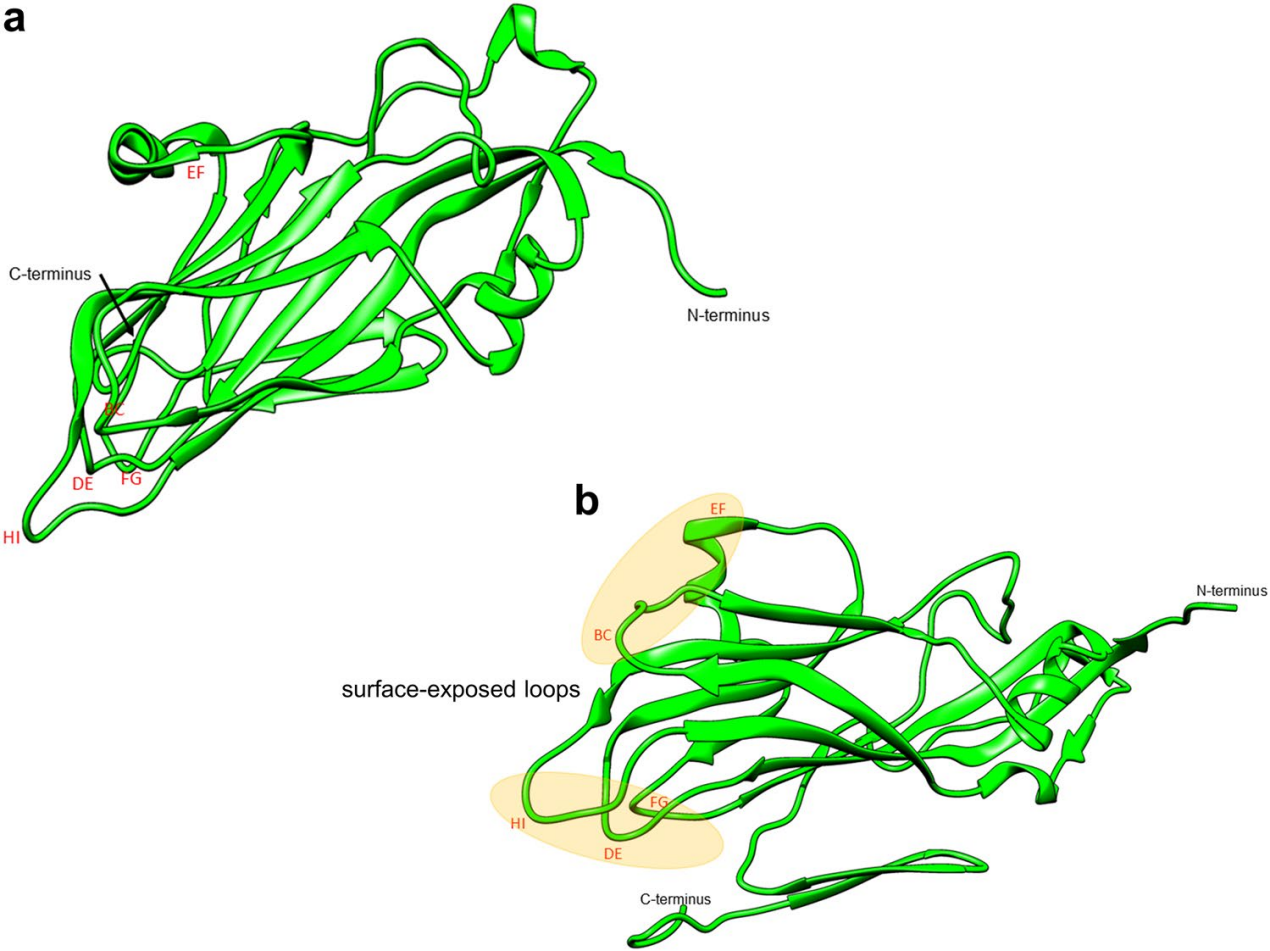
Ribbon diagram of VP2. The protein is shown in its original orientation within a protomer (**a**) and in a rotated position to show the surface-exposed loops of the protein that are highlighted in orange (**b**). The protein map is constructed from FMDV serotype O (PDB: 1FOD) [49] using the UCSF Chimera package [76]

VP2 consists of 218 or 219 amino acids, shorter than VP2 of other picornaviruses [40, 42] (Fig. 7). The N-terminal residues of the VP2 proteins of three adjacent pentamers are arranged around each threefold symmetry axis of the viral particle. A presumed calcium binding site, containing a conserved glutamic acid at position 6 of the protein, mediates an important ionic bond that supports the structural stability of the particle [41].

While unique amino acid substitutions have been described for different FMDV serotypes (see Table 2), there are parts of the protein where amino acid variations accumulate. Several amino acid exchanges are described for residues 78–80 and 130–131 for serotypes A and O [15, 50, 51, 54, 58, 63, 70, 71] as well as at position 77 for SAT2 [50]. While the residues 78–80 follow the BC loop at position 70–76, the residues 130–131 are part of the EF loop (position 130–137) [48]. These amino acids are displayed at the external surface of the viral particle [63, 70], constituting important antigenic sites of the virus [48]. The most frequently reported amino acid exchange is the replacement of the negatively charged anion glutamate at position 131 with a positively charged lysine (E131K). An extended receptor tropism has been described for serotype A and O viruses with amino acid exchanges at positions 78–80 and 130–131 [70]. Exchanges in this area seem to modulate the RGD-containing GH loop of VP1 by changing its spatial orientation, allowing the virus to either use HS or an unknown receptor for attachment to host cells [54, 58, 63, 70]. Furthermore, changes in this part of VP2 are often described to occur in combination with substitutions in VP1 [58, 63] during passaging in BHK cells [15, 71].

**Table 2:** Summary of reported amino acid substitutions in FMDV VP2

FMDV type	Virus protein	Amino acid substitutions	References
SAT2	VP2	I32V	[50]
O	VP2	R65H	[54]
A	VP2	F67L	[62]
O, A	VP2	G72S, D72N	[57, 69]
SAT1	VP2	Q74R	[50]
SAT2	VP2	M77T	[50]
O, A	VP2	C78Y, L78S	[63, 70]
A	VP2	E79A/G	[15, 58, 70]
O, A	VP2	L80Q, K80R	[54, 70]
A	VP2	E82A/K	[51]
SAT2	VP2	E96Q	[50]
A	VP2	W129R	[15]
O, C, A	VP2	C130Y, G130D, K130E	[63, 66, 70]
**A**	**VP2**	**E131K/G**	[15, 51, 58, 70, 71]
O	VP2	V132I	[63]
O	VP2	D133N	[72]
A	VP2	E134K/T134P	[15, 51]
O	VP2	E136G	[54, 57]
A	VP2	Q146E	[15]
A	VP2	T154M	[15]
A	VP2	N166D	[15]
SAT1, SAT2	VP2	Q170H, R	[50]
A	VP2	K172N	[15, 58]
O	VP2	K175R	[57]
C	VP2	A192T	[59]
C	VP2	G193S	[59]
SAT1	VP2	S196N	[50]
O	VP2	F214L	[57]
C	VP2	A277T/V	[73]

Where different amino acid exchanges at one position have been reported for different serotypes, they are separated by commas. If more than one exchange has been described for a serotype, these are separated by forward slashes. The most frequently reported amino acid exchanges are given in bold

Another well-described phenomenon for serotype A and O are amino acid exchanges at positions 133, 134, and 136 [13, 15, 51, 54, 57, 72]. This region lies within the αB helix of VP2 (residues 133–138) and is part of the depression that is used for HS binding [48]. Modifications at positions 170 to 175 have also been reported for serotype A and O viruses as well for SAT serotypes. The Q170H/R substitution in SAT1 and SAT2 viruses increases the positive charge around the threefold axis of the particle [50]. The K172N exchange described for a serotype A virus, on the other hand, reduced the positive charge of the region by substitution of a positively charged lysine with an uncharged asparagine [15, 58]. A third exchange, K175R in the GH loop of VP2, described for serotype O retained the positive charge at this position [57]. Variations in VP2 are often accompanied by amino acid exchanges in either VP1 or VP3. An overview of previously reported amino acid exchanges in VP2 is given in Table 2.

**Fig. 8:**
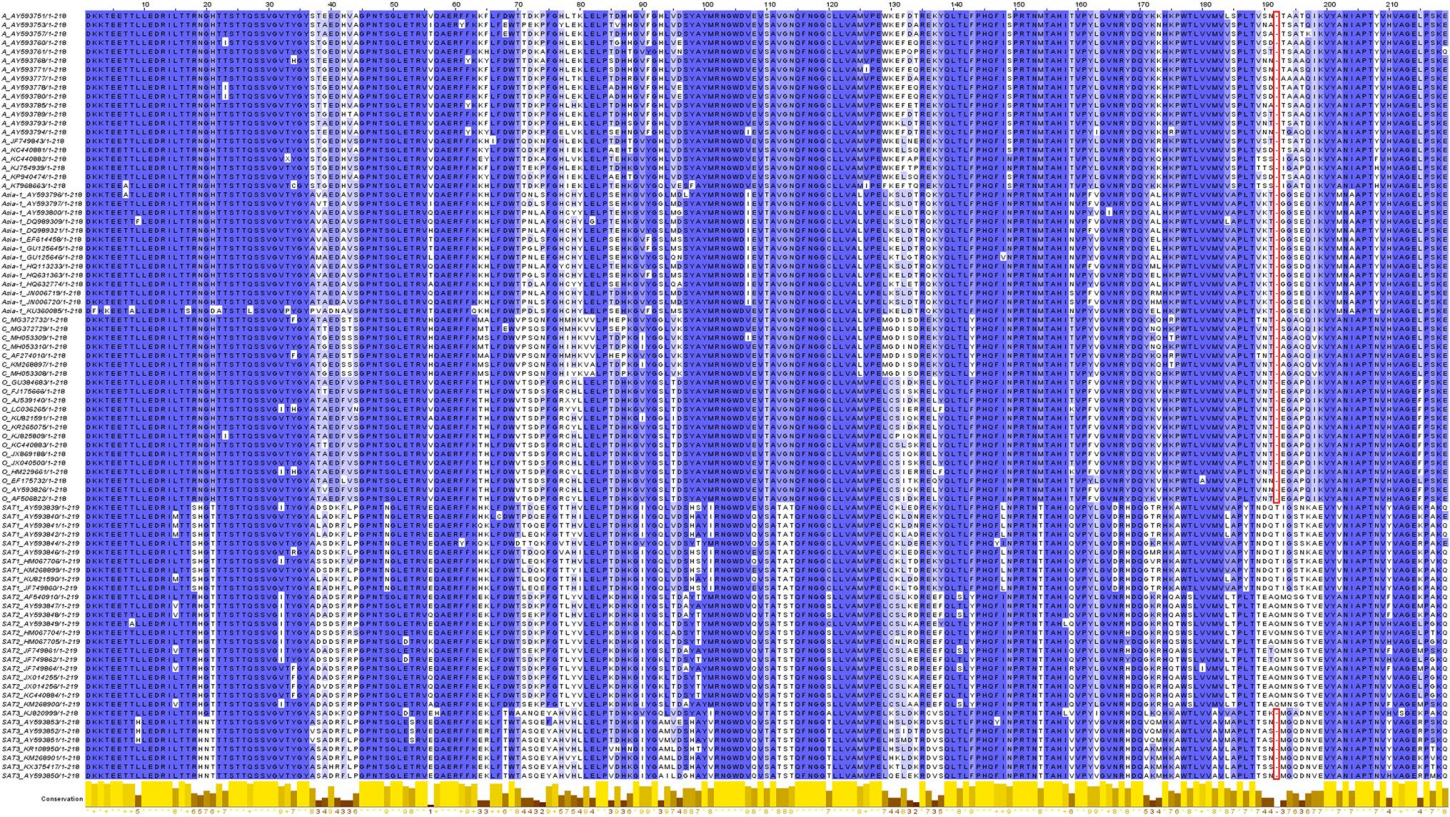
Protein alignment of aa sequences of all seven FMDV serotypes. Nucleotide sequences of the coding region of VP2 were retrieved from the GenBank database (https://www.ncbi.nlm.nih.gov/nucleotide/). Accession numbers are provided directly in the figure. Nucleotide sequences were translated into protein sequences by ExPASy [78]. Protein alignments and visualization were performed with Jalview [79]. Red boxes indicate positions where variations in the sequence lengths appear. The blue coloring indicates the conservation at a position, with darker shades of blue indicating higher degrees of conservation

A protein alignment of VP2 for FMDV isolates of all serotypes provides context for the described amino acid substitutions (see Fig. 8). While VP2 of the Eurasian serotypes and SAT3 consists of 218 amino acids, SAT1 and SAT2 have proteins of 219 aa in length. Overall, the alignment shows a high conservation of amino acids between serotypes, with variability mostly limited to positions 37–44, 56, 64–65, 70–80, 129–134, 173, and 189–199. As can be seen in the alignment, the position indices of substitutions before residue 192 are identical between all FMDV serotypes.

#### Virus protein VP3

**Fig. 9:**
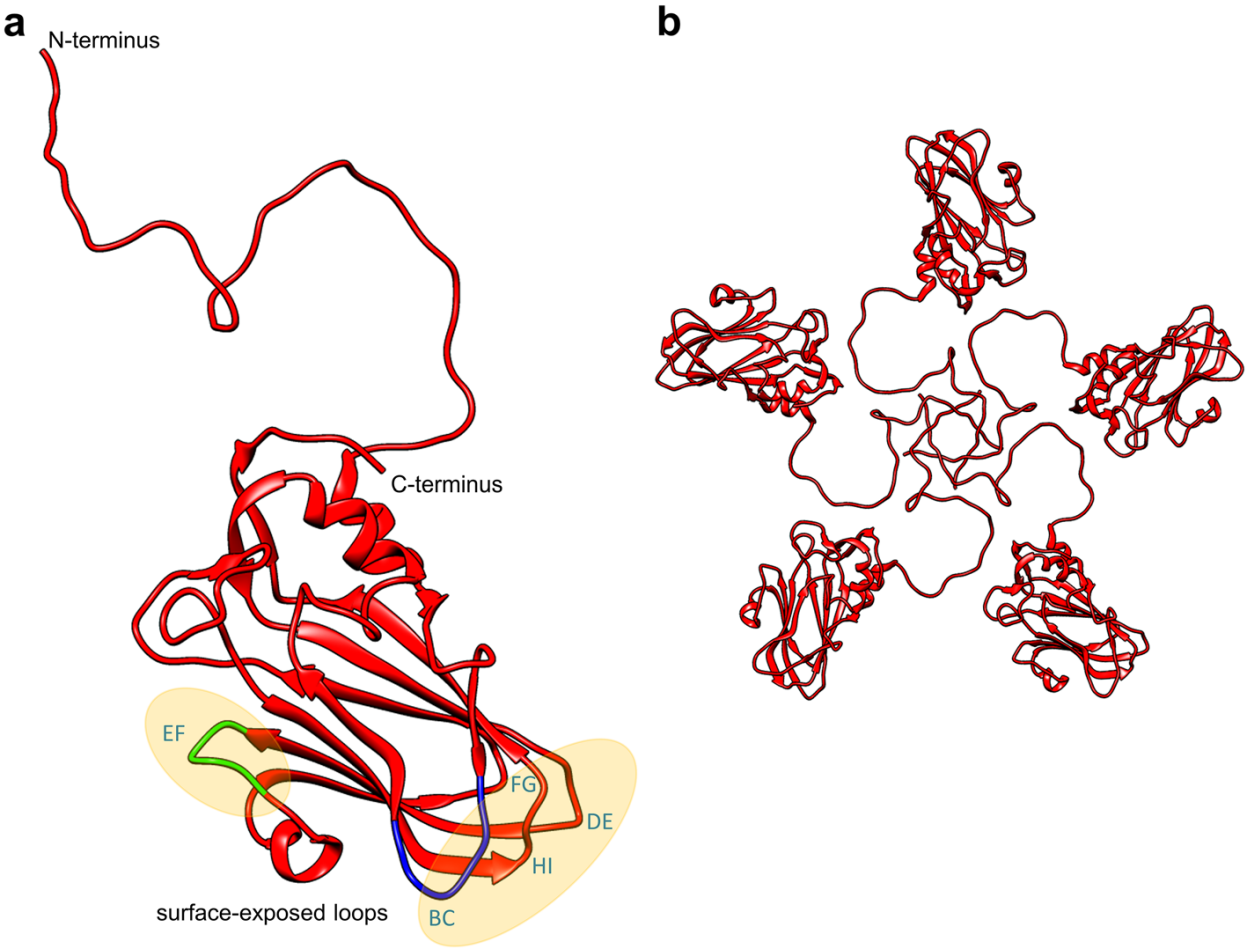
Ribbon diagram of VP3. The protein map is constructed from FMDV serotype O (PDB: 1FOD) [49] using the UCSF Chimera package [76]. **a** The variable BC loop is labeled in blue, while the EF loop is shown in green. The surface-exposed loops of the protein (highlighted in orange) are labeled according to Fry et al. [48]. **b** Linkage and channel formation by the N-termini of five VP3 proteins in a pentamer

VP3 is characterized by a high variability of 61% of its residues and a size of 219 to 221 amino acids [42]. Like VP2, VP3 is arranged around the threefold axis of the viral capsid. Furthermore, the N-termini of five copies of VP3 are interwoven around the fivefold axis to link the protomers that form a pentamer, while at the same time creating an axial channel that allows for the rapid permeation of small molecular entities such as caesium ions into the particle [40, 41]. This pore structure is highly hydrophobic due to the largely conserved amino acids phenylalanine, valine, and cysteine at positions 3, 5, and 7 of VP3, respectively [41] (Fig. 9).

Amino acid exchanges have been described for serotype C and SAT1 viruses at residue 7 (C7V) [59] and residue 9 (D9A/V) [50, 59], but these preserve the hydrophobic character of this region. A cluster of substitutions near the N-terminus of VP3 was reported again for type C (N13H, M14L, A25V) and SAT viruses (T43S, Q49E) [50, 53, 59, 66] in connection with an extended receptor tropism after serial passaging of the virus in BHK cells. One of the most variable areas of the protein lies between residues 55 and 88 [42], which are part of the HS binding site of the virion [48]. Several publications describe substitutions toward a positive charge on position 56 for serotype A [51, 52] and O [13, 72, 74], structurally the βB “knob” of VP3 that forms one of the walls of the HS binding depression [48]. Close to that position, at residue 59, the exchange of negatively charged glutamate with a positively charged lysine has been reported for serotype Asia-1 during the adaptation of the isolate to grow in BHK suspension cells [33]. This residue is located at the loop downstream of the B1 strand, and is a part of the HS binding site on the virus particle [48]. Residues 84–88 shape the bottom of the indentation [48] but amino acid exchanges in this region do not always result in the expected acquisition of positively charged residues (H85Q [52], H85R, M86V, M86T [15]).

A highly variable region of VP3 is proposed to be at positions 130–140. Substitutions toward a positive charge at the surface-exposed loops of the βE–βF part of the protein (T129K, E135K) seem to be advantageous for SAT serotypes to adapt to cell culture [50]. Other amino acid exchanges within this variable region were described for a serotype A strain adapting to BHK suspension cells (E138G, K139E) [15] and when adapting a SAT virus to adherent BHK cells (D132N) [50]. Another “adaptive hot-spot” lies between positions 173 and 180, where a multitude of substitutions has been reported in the course of cell culture adaptation of virus isolates of various serotypes (A, O, C, and SAT1) [15, 50, 51, 53, 57, 62, 66]. Amino acid exchanges at the C-terminus of the protein are also quite common among Eurasian and SAT serotypes but there are no detailed descriptions of the effect of these mutations [15, 50, 53, 56, 66]. Because the C-terminus is located at the outer surface of the virus particle, exchanges that support adaptation to culture conditions are likely to occur in this area. A summary of previously reported amino acid substitutions in VP3 is shown in Table 3.

**Table 3:** Summary of reported amino acid substitutions in FMDV VP3

FMDV type	Virus protein	Amino acid substitutions	References
C	VP3	C7V	[59]
C, SAT1	VP3	D9A, V	[50, 59]
C	VP3	N13H	[59]
C	VP3	M14L	[59]
C	VP3	A25V	[53, 66]
SAT2	VP3	T43S	[50]
SAT2	VP3	Q49E	[50]
**A, O**	**VP3**	**C56R, H56R**	[13, 51, 52, 72, 74]
Asia-1	VP3	E59K	[33]
A	VP3	K76E	[51]
A	VP3	H85Q/R	[15, 52]
A	VP3	M86V/T	[15]
A	VP3	S92P	[15]
SAT2	VP3	T129K	[50]
SAT2	VP3	D132N	[50]
SAT1	VP3	E135K	[50]
A	VP3	E138G	[15]
A	VP3	K139E	[15]
SAT2	VP3	E148K	[50]
A	VP3	T150A	[15]
C	VP3	E173K	[53, 66]
O	VP3	A174S	[54]
A	VP3	D174G, T174K, V174A	[51, 62]
SAT1	VP3	E175K	[50]
A	VP3	A176P	[15]
A	VP3	E177A	[15]
C	VP3	C178S	[66]
SAT1	VP3	A180V	[50]
SAT1, SAT2	VP3	D192T/Y, P192T	[50, 56]
A	VP3	T199A	[15]
SAT1	VP3	S203T	[50]
SAT1	VP3	S217I	[50, 56]
C	VP3	Q218K	[53, 66]
A, SAT1	VP3	R219C, S219L	[15, 50]

Where different amino acid exchanges at one position have been reported for different serotypes, they are separated by commas. If more than one exchange has been described for a serotype, these are separated by forward slashes. The most frequently reported amino acid exchanges are given in bold

**Fig. 10:**
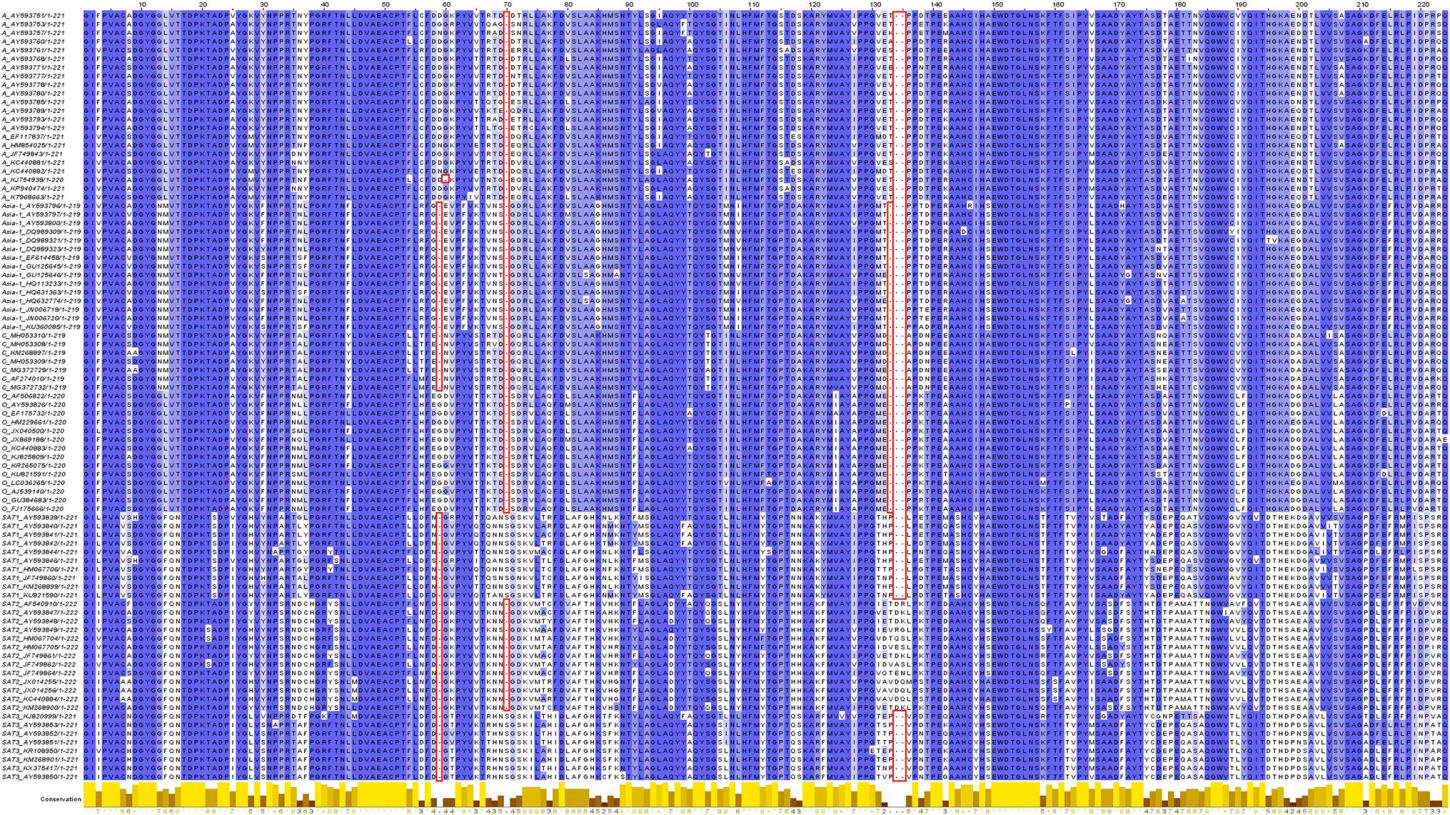
Protein alignment of aa sequences of all seven FMDV serotypes. Nucleotide sequences of the coding region of VP3 were retrieved from the GenBank database (https://www.ncbi.nlm.nih.gov/nucleotide/). Accession numbers are provided directly in the figure. Nucleotide sequences were translated into protein sequences by ExPASy [78]. Protein alignments and visualization were performed with Jalview [79]. Red boxes indicate positions where variations in the sequence lengths appear. The blue coloring indicates the conservation at a position, with darker shades of blue indicating higher degrees of conservation

The protein alignment for VP3 shows 219 aa for serotypes Asia-1 and C, 220 aa for serotype O and 221 aa for SAT1 and SAT3. The highly variable serotype A includes isolates with 220 aa and 221 aa. Contrary to previously published data [42], VP3 of the SAT2 isolates used for the alignment was 222 aa in length. The different lengths of VP3 are due to deletions at positions 59, 70, and 133–135 (see Fig. 10).

#### Virus protein VP4

**Fig. 11:**
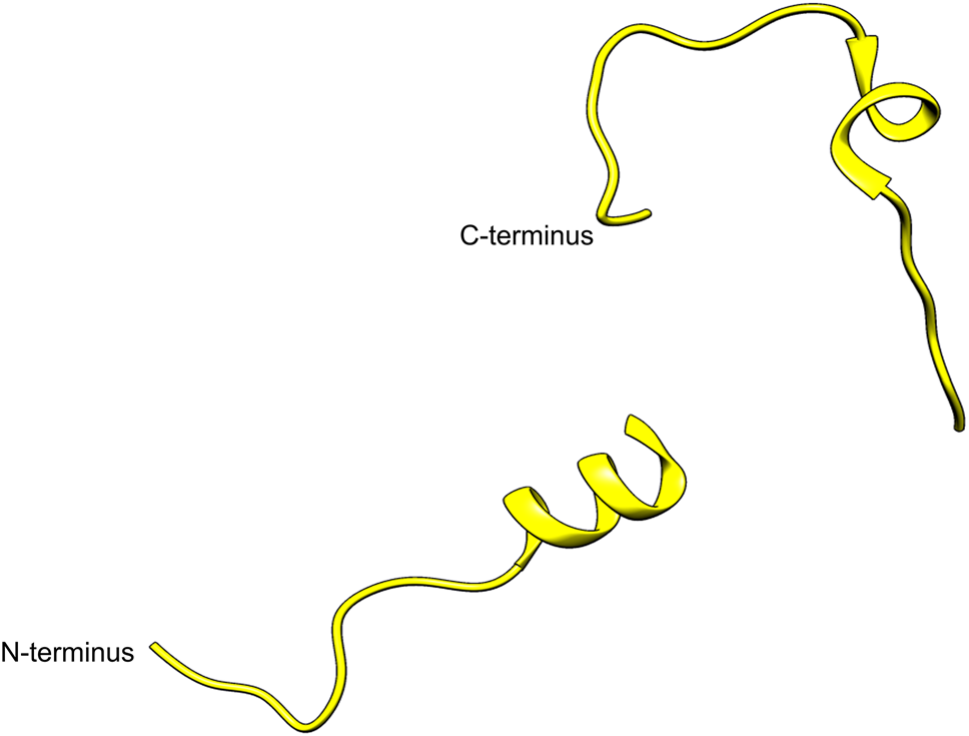
Ribbon diagram of VP4. The gap is due to the unresolved 3D structure of residues 40–64. The protein map is constructed from FMDV serotype O (PDB: 1FOD) [49] using the UCSF Chimera package [76]

VP4 is the most conserved FMDV protein with only 29% variable amino acids [42]. It is a small, highly hydrophobic protein, located on the inside of the capsid [75]. With 85 residues overall, FMDV has the longest VP4 protein among the picornaviruses, but the 3D structure of residues 1–15 and 40–64 is still unresolved [40, 41]. Unusual for picornaviruses, the amino acid chain of VP4 forms a helix of three turns, with the myristoylated N-terminus close to the fivefold axis and the C-terminus close to the threefold axis of the particle [40, 41] (Fig. 11).

Amino acid exchanges in this protein have been described very rarely. Single substitutions were reported for serotype A and serotype O viruses that occurred sporadically [15, 62] or in combination with other amino acid exchanges in other proteins [58] (see Table 4).

**Table 4:** Summary of reported amino acid substitutions in FMDV VP4

FMDV type	Virus protein	Amino acid substitutions	References
O	VP4	A8S	[54]
A	VP4	S15A	[15]
O	VP4	T52A	[62]
A	VP4	S77G	[58]

## Conclusions

At best, the adaptation of a field isolate of FMDV to grow in cell culture is laborious and time-consuming, if it is possible at all. Virus strains that do not seem to be able to grow in a particular cell line as well as cell lines that appear to be resistant to certain strains are commonly described problems. This is of particular concern for the development of vaccines to newly emerging virus lineages. Long lead times from virus isolate to vaccine can delay the implementation of effective control programs, and high antigen yields in production cells are essential for affordable vaccines. A rational approach to cell culture adaptation that combines prior knowledge of common adaptive mutations and reverse genetics techniques is urgently required. The ability to engineer recombinant viruses with enhanced growth properties in cell culture can be of great benefit to both the producers and users of FMD vaccines.

Beyond a simple list of observed changes during cell culture adaptation, it may be useful to look for patterns in adaptive mutations. While changes in VP1 can occur and presumably be effective in isolation, adaptive mutations in VP2 were usually seen in combination with amino acid exchanges in VP1 or VP3. The reason for this is unclear, but a possible explanation may lie in the spatial arrangement of the proteins. VP1 has the largest number of surface-exposed residues, while VP2 and VP3 have a larger share of residues involved in forming the virus particle itself, and unbalanced amino acid exchanges in these regions might therefore compromise the integrity of the particle. Many residues in VP1, on the other hand, are free to change without restraint and are more likely to have an immediate effect on virus–cell interactions due to their prominence on the surface on the virion. More research into these patterns is required to identify minimum sets of mutations that maximize virus replication in culture while at the same time preserving the antigenic profile and the stability of the viral particles.

This review focused on amino acid exchanges in the capsid proteins in the context of adaptation of the virus to cell culture. Obviously, synonymous and non-synonymous mutations of the FMDV genome can have other implications—resistance to degradation by low pH or high temperature, viral population kinetics during acute or persistent infection, and many more—but these are outside of the scope of this review.

## Electronic supplementary material

Below is the link to the electronic supplementary material.

Supplementary material 1 (FASTA 21 kb)

Supplementary material 2 (FASTA 20 kb)

Supplementary material 3 (FASTA 21 kb)
